# On the Recent Progress and Future Prospects of Practical Medicine

**Published:** 1846-07

**Authors:** Elisha Bartlett

**Affiliations:** Professor of Medicine in the University of Maryland


### PART THIRD.

### CONTRIBUTIONS TOWARDS THE ADVANCEMENT

### OF THE

### Natural History and Treatment of Diseases.

---

### I. ON THE RECENT PROGRESS AND FUTURE PROSPECTS OF PRACTICAL MEDICINE.

By Elisha Bartlett, m.d.,
Professor of Medicine in the University of Maryland.

[The following observations are so excellent in themselves, and in such perfect accordance with the views recently promulgated in this Journal, on the subject of therapeutics, that we feel it a duty to lay them before our readers. They are extracted from the author's excellent work on the 'Philosophy of Medicine,' which was briefly noticed in our Twentieth Volume, p. 140. The observations are the more important because we have reason to know from other sources that they express the sentiments of the best and most experienced men in the United States. This return to a milder, more rational, and more natural system of practice, has been mainly brought about, we believe, by actual experience of the vast mischief produced by the heroic treatment formerly in vogue, which treatment was carried to an extent of boldness, or violence, unknown even in England. We know, on the best authority, that, not many years since, it was the practice of a professor of medicine, at one of the American Universities, to recommeud and to prescribe calomel in *tablespoonfuls!* Even in this book Dr. Bartlett, reprobating this system, tells us: "It [calomel] is constantly administered—on all occasions—in all diseases—and in all their stages. It has, literally, in some instances, been made an article of *daily food*—sprinkled upon buttered bread, and mixed with it before baking! I suppose it is no exaggeration to say, that there is more calomel consumed in the valley of the Mississipi and its tributaries, than in all the world beside."]

"The history of practical medicine, especially, during the last twenty-five years, and a right appreciation of its character, and the conditions and means of its progress, furnish us with very positive assurance that many of its most important laws will gradually, but steadily and certainly, be carried forwards to their entire and final establishment. The foundations of many of these laws,—and of those, too, most difficult of determination,—have been already broadly and securely laid; and although many years must elapse, amidst earnest, unremitting, and conscientious toil, before these laws can be *definitively* and *fully* settled, it is not possible, in the nature of things, that we can be deceived, or disappointed, in this consummation, so devoutly to be wished.

The minute and thorough study of diseases, in all their aspects, phases, and relationships, which is now prosecuted, with so much zeal and fidelity, cannot fail of leading to the result of which I have spoken. The great laws of pathology and its relations,—of etiology, and therapeutics,—are sure to be ascertained; each successive year will add something to their development, in the steady accumulation of legitimate and authentic materials, and in their disposition and analysis, so that, in the end, the *entire natural history of diseases* will be made out and written.

"In this progress of medical science, which we thus confidently anticipate, some of its branches will take precedence of others. Diagnosis, for instance, will be in advance of therapeutics; and this for two reasons. In the first place, the elements of the former are fewer, and less complex in their relationships, than those of the latter; and in the second place, diagnosis is an *essential pre-requisite* of therapeutics. These are amongst the reasons why improvements in the treatment of disease, especially for the last twenty-five years, have not kept pace with the advances, which have been made in our knowledge of disease itself. After our knowledge of pathology, and our nosological diagnosis growing out of this, have reached their highest attainable point of accuracy and positiveness, there is still left an almost interminable field of investigation, in the study of the relationships between the morbid condition, thus ascertained, and the substances and agencies in nature, which can in any way affect or influence this condition. Let us look, for a single moment, at the extent and the complexity of these relationships. They are almost infinite. Look at any single disease, even of the simplest and best settled character; and let us suppose that all its elements, as far as this is possible, in the nature of things, have been accurately ascertained. Before our therapeutical knowledge of this disease can be said, in literal strictness, to be *complete,* we must know the effects and influences, which *all the substances and agencies in nature are capable of producing upon it;* and we can know this only by direct observation of the effects themselves. We must know how it will be modified by each and all of the different vegetable productions of the earth; by each and all of the mineral substances, in their manifold forms of chemical combination; by changes of temperature, and other meteorological conditions; by light; by electricity; by food; by drink; by exercise; by the state of the mind, and so on. The doctrine, thus stated, sanctions the constant introduction and trial of new remedies; since until any given substance is tried we do not and cannot know what properties of a remedial nature it may be endowed with. *All* substances, in their remedial characters, were once new; calomel, antimony, opium, Peruvian bark, were once, and some of them not very long ago, new remedies; and any philosophy that would reject the trial of a remedy now, *because* it is new, would of course have rejected the trial of these on the same ground. But, let me say, there is no man, anywhere, who regrets more sincerely than I do the multiplication which is constantly taking place of the so called articles of the materia medica. There is probably no man more entirely sceptical in regard to their alleged properties and virtues than I am. There is no man who has been in the habit of using a smaller number of them. There is nothing in the whole range of medical history, which shows so miserable a logic, and so false a philosophy, as the introduction of this multitudinous assemblage of new remedies, *with the properties which are so confidently assigned to them.* But then the fault and the error are,—not that the remedies are new,—but that the evidence of their value and efficacy is so utterly wanting. My own opinion is,—an opinion founded upon the history and experience of all the past,—that the number of substances endowed with active, and peculiar or characteristic, remedial properties is small. But whether this number is small or large can be determined only by observation and experience, *or trial.*

The true course of the philosophical physician is,—not to reject the medicine *because it is new,* but for the reason, abundantly sufficient in regard to nineteen twentieths of the articles of the official pharmacopœias, that there is no satisfactory evidence that it is worth anything; and one of the most certain and beneficial results of a correct medical philosophy will be the final expulsion and banishment of these aliens and impostors from the domain of our science.

"Now, when it is remembered, that these substances and agencies are, many of them, acting together,—that it is exceedingly difficult, in many cases, to separate the influence of one from that of another in our own endeavours to estimate the real agency of each; and, furthermore, that the elements of the disease itself, so far at least as its therapeutical relationships are concerned, are more or less fluctuating and changeable,—it must at once be seen how true it is, as I have already said, that positive therapeutical knowledge is more difficult of attainment than any other in the entire circle of medical science.

"But, notwithstanding all these formidable and inherent difficulties, this knowledge has made, within the period of which I am speaking, great and positive advances. The effects of many remedies are much better understood, and their value much more accurately appreciated than formerly. And I believe, that hereafter, this department of our science and art is destined to a more rapid and positive advancement, when compared with the other departments, than has hitherto been its lot. The first essential condition of this advancement,—the accurate and positive diagnosis of disease,—has to a good degree been fulfilled. The first element in the problem to be solved has been ascertained; and we accordingly find, that the attention of many of the best minds in the profession is now turning in this direction. This is the natural course cf events. The seat, the character, the regular march, and the tendencies of the disease, having been first ascertained, the next thing to be done is to find out the best methods of preventing, of modifying, and of curing it. This is what many of the great pathologists of the present day are actively and zealously engaged in endeavouring to do. This is the great mission which now lies immediately before us; this is to constitute the great work of the next and succeeding generations.

"I should be doing great injustice to my subject, if I did not mention, as prominent amongst the therapeutical improvements of the last quarterof a century, the change which has been gradually taking place, in the use of violent and dangerous remedies. I am inclined to regard this change as one of the greatest blessings which modern medical observation has conferred upon the human race; and it is but fair to admit, that absurd as the *system* of homœopathy is, and unsupported as its pretensions are, so far as its *peculiar treatment* of disease is concerned; it has, nevertheless, done great good *by its practice,* —its scrupulous adherence to a strict regimen, and its avoidance of all injurious remedies,—in the furtherance of this revolution. 'It has been sarcastically said, that there is a wide difference between a good physician and a bad one, but a small difference between a good physician and no physician at all; by which it is meant to insinuate, that the mischievous officiousness of art does commonly more than couterbalance any benefit derivable from it.' (*Sir Gilbert Blane.*) The conviction has been steadily gaining ground, and spreading itself abroad in the medical community, not only that *heroic* remedies, as they are called, are often productive of great mischief, and should never be lightly or questionably used; but that in very many cases of disease, *all medicines,* using this word in its common signification, are evils; and that they may be dispensed with, not merely with negative safety, but to the actual benefit of the subjects. The golden axiom of Chomel, that it is only the *second* law of therapeutics *to do good,* its *first* law being this—*not to do harm*—is gradually finding its way into the medical mind, preventing an incalculable amount of positive ill. The real agency of art is more generally appreciated than formerly; and its arrogant pretensions much more truly estimated and understood. It is coming every day to be more clearly seen, that perhaps its most universal and beneficent function consists in the removal and avoidance of those agents, the action of which is to occasion or to aggravate disease; thus giving the recuperative energies of the system their fullest scope and action, and trusting to them, when thus unembarrassed and free, for the *cure* of the disease. 'This, I apprehend, is so well understood among well educated physicians, that the word *cure*, as applied to themselves, is proscribed as presumptuous, and rarely, I believe, escapes the lips of any practitioner, whose mind is duly tinctured with that ingenuous modesty which characterizes the liberal and correct members of the profession.' (*Sir Gilbert Blane.*)

" It is melancholy to think what an enormous aggregate of suffering and calamity has been occasioned by a disregard of the axiom which I have quoted. Our means for the direct removal of disease are limited in extent, but it is not so with our power to augment and to cause it; this is unlimited. Difficult as it may be to cure, it is always easy to poison and to kill. We may well congratulate ourselves and society, that the primary and fundamental truths, of which I have been speaking, are finding their right position, and producing their legitimate results; and that long abused humanity is likely, at no very remote period, to be finally delivered from the abominable atrocities of wholesale and indiscriminate *drugging*.

" I cannot forbear remarking, by way of parenthesis, that this evil, in addition to the many others which I have already had occasion to enumerate, has been greatly aggravated, and in many instances wholly produced, by the influence of à priori medical doctrines. The whole history of medicine will show that the most flagrant abuses of this character have always been the direct results of these mischievous influences."

